# Eye, Ear, Nose and Throat

**Published:** 1893-06

**Authors:** 


### EYE, EAR, NOSE AND THROAT.

EDITED BY DR. ARTHUR G. HOBBS, ASSISTED BY DR. GEORGE BROWN.

### THE ELECTRO-MAGNET.

Dr. William Ellery Briggs reports twenty-two cases in which the electro-magnet was used to remove pieces of steel that had entered the eye.

In fifteen of the cases the steel was withdrawn by the magnet, five of these cases obtained perfect vision after the operation, five resulted in traumatic cataract, two in total blindness, two had perception of light, and one vision equal to 20-200. In the seven cases that the magnet failed to remove the steel, four eyes were enucleated, two recovered perfect vision, and one was lost sight of. Four pieces of steel were found in the iris, one between the iris and lens, sixteen in the vitreous, and one in the lens and vitreous. The length of time intervening between the time the injury was received and the operation ranged from a few hours to three weeks. —*The Occidental Medical Times, April,* 1893.

NOTE: Hobbs has reported thirteen cases in which he has used the electro-magnet in similar cases. In eighteen of these the steel was successfully removed. He uses the ordinary instrument manufactured by Meyrowitz, applied to his galvano-cautery battery governor, which he gets from an alternating current introduced into the office by a street wire. Some of these operations have resulted successfully when any other means would have failed and an enucleation would have been unavoidable. G. B.

---

### THE TONSILS AND THEIR CHRONIC ENLARGEMENT.

Dr. F. W. Blake (*Columbus Medical Journal*) says: The conclusions I would draw from my consideration of the subject are:

1. The precise functions of the tonsils are not determined, but from experimental research and clinical observation we may assign to them absorptive powers, or at least peculiar vulnerability to septic influences; hence antiseptic measures are indicated both in the prophylaxis and treatment of acute disorders.

2. That the evils arising from enlarged tonsils are not solely transitory; but that in the outgrowing of their local effects other conditions of disability, both physical and mental, may be induced, the effects of which may be widespread and permanent.

3. That the loss of the organ entails no evil effects.

4. That chronic enlargement is usually the evidence of diathetic influence, and constitutional treatment is of value.

5. That whenever the condition of the tonsils is such as to invite infection or to interfere with any bodily function, it is sufficient cause to advise their excision, though other procedures for their reduction may be properly elected.

6. That excision by the tonsillotome is to be considered in the case of children as entirely free from dangerous hemorrhage, but in the case of adults the snare is the safer instrument. In all cases, however, provision should be made for combating the possible hemorrhage.

7. That in the operation of excision, while there is no future advantage in leaving part of the tonsil, there is also no demand for total extirpation. The partial removal is often more feasible and safe, and in its effects all-sufficient.—*The American Lancet, April*, 1893.

---

### EXTRACTION OF BITS OF STEEL OR IRON FROM THE CORNEA.

It is not infrequently (*Va. Med. Monthly*) the case where small bits of iron or steel have been imbedded in the cornea—especially when they have come from a heated piece of metal—that after their removal the corneal wound shows no tendency to heal rapidly, and that the irritation and discomfort of the wound continue. According to Dr. John Dunn, Chief Clinic of the Richmond, Va., Eye, Ear and Throat Infirmary, examination of these wounds with artificial light will most frequently reveal at their bottom what at first sight appears to be small bits of the original foreign body. They are not so, however; but are either small areas representing the action of oxidizing iron on the corneal tissue, or are partly burnt corneal tissue. These small areas act as foreign bodies, and the corneal wound shows little tendency to heal as long as they remain. Often, when the corneal wound is deep, they require considerable skill to detach them. At times they may be scraped away; while in other cases, after detaching them, they have to be cut off with a small pair of scissors. They should be carefully sought for after the extraction of every piece of steel.—*The Canada Lancet, May*, 1893.

